# Acquired Nasopharyngeal Stenosis Correction Using a Modified Palatal Flaps Technique in Obstructive Sleep Apnea (OSA) Patients

**DOI:** 10.3390/ijerph17062048

**Published:** 2020-03-19

**Authors:** Giovanni Cammaroto, Luigi Marco Stringa, Luca Cerritelli, Giulia Bianchi, Giuseppe Meccariello, Riccardo Gobbi, Giannicola Iannella, Giuseppe Magliulo, Henry Zhang, Ahmed Yassin Baghat, Francesco Galletti, Stefano Pelucchi, Francesco Stomeo, Muawya Bani Younes, Mohamed AlAjmi, Andrea De Vito, Claudio Vicini

**Affiliations:** 1 Head and Neck Department, ENT & Oral Surgery Unit, G.B. Morgagni, L. Pierantoni Hospital, 47100 Forlì, Italy; giuseppe.meccariello@auslromagna.it (G.M.); dr.riccardogobbi@gmail.com (R.G.); giannicola.iannella@uniroma1.it (G.I.); fgalletti@unime.it (F.G.); dr.andrea.devito@gmail.com (A.D.V.); claudio@claudiovicini.com (C.V.); 2 Department of Otolaryngology, University of Messina, 98100 Messina, Italy; 3 Department of Otolaryngolgy, Head and Neck Surgery, University of Ferrara, 44121 Ferrara, Italyluca.cerritelli.bo@gmail.com (L.C.); giuwhites91@gmail.com (G.B.); stefano.pelucchi@unife.it (S.P.); stmfnc@unife.it (F.S.); 4 Department of ‘Organi di Senso’, University “Sapienza”, 00100 Rome, Italy; giuseppe.magliulo@uniroma1.it; 5 Department of Otolaryngology, Head and Neck, Royal London Hospital, Whitechapel road, E11BB London, UK; drhenryzhang@gmail.com; 6 Department of Otorhinolaryngology, Alexandria University, Alexandria 22206, Egypt; ahmedyassinbahgat@gmail.com; 7 Unit of Otolaryngology, Head and Neck Surgery, Sara Speciality Hospital, Amman 11118, Jordan; Chief.medical.officer@sarahspecialtyhospital.com; 8 Department of Otolaryngology, Head and Neck Surgery, AL Nahdha Hospital, Muscat 111, Oman; dr.mohamed8619@gmail.com

**Keywords:** nasopharyngeal stenosis, pharyngeal surgery, OSA, nasal obstruction

## Abstract

*Background*: Acquired nasopharyngeal stenosis is a rare and heterogeneous pathological condition that has different causes, generally resulting as a complication of a pharyngeal surgery, especially in patients affected by obstructive sleep apnea (OSA). Different approaches have been proposed for the treatment of nasopharyngeal stenosis but a unique and standardized management has not yet been presented. The aim of our paper is to evaluate the efficacy of our surgical technique, describing its steps and results with the aim to consider it as a possible solution for the treatment of this condition. *Methods*: This is a retrospective cohort study. Eight patients (mean age 27.25 years old (yo), range 8–67 yo; Male/Female ratio 4/4; mean body mass index (BMI) 26.1) affected by OSA (mean apnea hypopnea index (AHI) before OSA surgery was 22.1) and acquired nasopharyngeal stenosis as a consequence of different pharyngeal surgeries were treated with our modified approach in the Department of Otolaryngology, Morgagni Pierantoni Hospital, Forlì, Italy. Resolution of stenosis and complication rate were the main outcome measures. *Results*: Complete resolution of the stenosis was achieved in all cases and no complications were recorded at three weeks, six months, and 2 years follow-up. *Conclusions*: Our technique appears to be a promising method for the management of nasopharyngeal stenosis in OSA patients. However, further studies comparing different techniques and reporting on larger series and longer follow up time are needed to prove the efficacy of the proposed technique.

## 1. Introduction

Nasopharyngeal stenosis is an uncommon acquired pathological condition characterized by the reduction and, sometimes, by the complete obstruction of the nasopharyngeal isthmus [1].

This particular condition is usually an iatrogenic entity following therapeutic procedures such as: tonsillectomy, adenoidectomy, uvulopalatopharyngoplasty (UPPP), and radiotherapy.

Unfortunately, there is no clear data in literature about the prevalence of this complication in patients treated with UPPP. On the other hand, a study of Imperatori et al. reported on three cases of nasopharyngeal stenosis in a series of 100,000 cases of tonsillectomy and adenoidectomy [2].

Many risk factors seem to participate, including an excessive removal of mucosa, re-interventions, keloid diathesis, presence of a concomitant pharyngeal inflammation, and an excessive use of electrocautery during the surgery. In some rare cases, nasopharyngeal stenosis is a possible evolution of inflammatory reactions in patients affected by sarcoidosis, oropharyngeal localization of syphilis [3,4,5,6,7].

An exuberant fibrotic tissue is at the base of this condition, leading to scar contraction, fusion of the soft palate and the posterior tonsillar pillars to the posterior pharyngeal wall with the reduction of the free lumen.

The consequences due to this entity include dyspnea, dysphagia, poor weight gain, rhinolalia, velopharyngeal insufficiency, otitis media, anosmia, and obstructive sleep apnea (OSA) [4,6].

Several surgical techniques have been proposed in literature, some of them not intuitively replicable and with partially satisfactory outcomes, but the scientific community has defined no gold standard treatment yet. One of the most used and efficient surgical procedures is the one proposed by Toh et al. [8] consisting of the transposition of a bi-valved palatal flap in order to increase the volumes of the upper aerodigestive tract.

Here, we report on our surgical variation in a series of patients affected by OSA and nasopharyngeal stenosis as a consequence of different surgeries. The purpose of our technique is to reduce the incidence of post-operative complications such as velo-pharyngeal insufficiency by splitting the soft palate into two flaps and suture the muscular layers of both flaps anteriorly and laterally in order to increase the retro-pharyngeal volume. The aim of our paper is to propose a replicable, less-invasive, and effective procedure.

## 2. Materials and Methods

### 2.1. Patients and Methods

This preliminary retrospective study was conducted at the Otolaryngology, Head and Neck Surgery Department, G.B. Morgagni-L. Pierantoni Hospital, ASL of Romagna, Forli, Italy, starting from February 2014 until December 2017. Prior to conduction of the study, informed detailed consent was taken from patients.

Patients were enrolled in conformance with the following inclusion/exclusion criteria:

(a) Inclusion Criteria: Patients affected by OSA diagnosed with post-surgical (at least 1 year after surgery) mild to moderate (more than 50–75% reduction of lumen, subjectively measured by clinicians; stenosis was graded as mild (50%) when the lateral aspects of the palate adhered to the posterior pharyngeal wall, moderate (75%) when there was circumferential scarring with a small central opening that was 1–2 cm in diameter) nasopharyngeal stenosis evaluated by means of upper airways endoscopy and oral cavity exploration. Main indication for surgery was correction of stenosis.
(b) Exclusion Criteria: Patients with severe medical illness, patients with severe craniofacial anomalies affecting airway, patients with limited mouth opening (inter-incisive distance < 1.5 cm), and patients unfit for general anesthesia (American Society of Anesthesiologists score (ASA) > 3).

### 2.2. Outcome Measures

(a) Subjective measure of nasopharyngeal stenosis by means of upper airways endoscopy and oral cavity exploration.
(b) Baseline, 3 weeks, 6 months, and 2 years Visual Analog Scale (VAS 0–10) for the evaluation of nasal breathing performance, dysphagia, and dysphonia was administered (0 “no complaints” to 10 “severe complaint”).
(c) Baseline and 2 years follow-up polygraphy evaluating the apnea-hypopnea index (AHI) were performed. All the sleep studies were carried out by means of a Polymesam Unattended Device 8-channel, reviewed and scored by the same expert in sleep medicine according to the American Academy of Sleep Medicine Guidelines [9].

### 2.3. Surgical Technique

**Figure 1:**
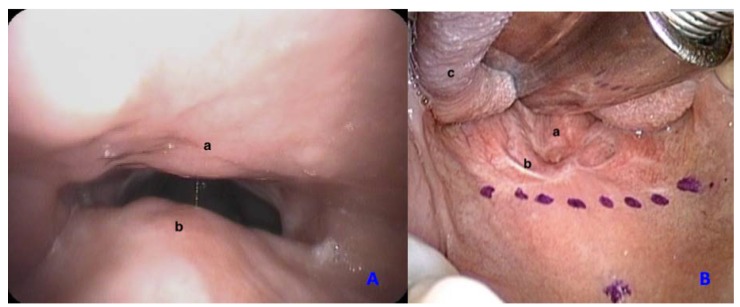
Pre-operative vision of nasopharyngeal stenosis from a nasopahryngeal (**A**) and oral (**B**) perspective. a—posterior pharyngeal wall, b—soft palate, c—tongue.

**Figure 2:**
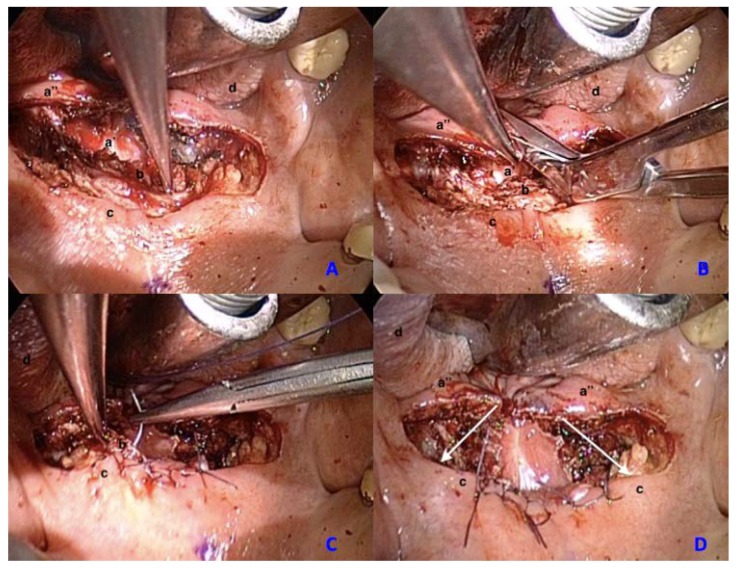
Surgical steps: dissection of muscular layer (**A**), splitting of nasopharyngeal mucosal flap (**B**), suture of the muscular layer to the oral mucosal layer (**C**), eversion of the left and right palatal flaps anteriorly and laterally (**D**). a’—nasopharyngeal mucosa inner face, a’’—nasopharyngeal mucosa external face, b—muscular layer, c—oral mucosal layer, d—tongue.

The surgical procedure is performed under general anesthesia with oral endotracheal intubation, thus the patient is placed in the supine position and Kilner-Doughty mouth gag is used to obtain the exposure of the oropharynx. Before starting the procedure, the operative site is infiltrated with Bupivacaine Hydrochloride 0.5% to control bleeding and reduce post-operative pain. Throughout the procedure, only cold steel instruments are used and a selective hemostasis is performed with bipolar cautery. A horizontal incision of the soft palate is performed from one anterior tonsillar pillar to the other (Figure 1). Subsequently, the dissection of the muscular layer is performed until the nasopharyngeal mucosal layer of the soft palate is reached. The nasopharyngeal mucosal flap of the soft palate is then split vertically to obtain a right and left palatal flap. An accurate suture is drawn up with the aim of opening the stenosis, reducing as much as possible the exposure of sub-mucosal tissue, which is reported as one of the principal factors of surgical failure. For this reason, interrupted 4-0 vicryl sutures are used in order to tie the muscular layer anteriorly to the oral mucosal layer of the soft palate and at the same time pull both lateral palatal flaps anteriorly and laterally to the oral palatal mucosa (Figure 2). In addition to this, the patients are maintained on a soft and cold diet for two–three weeks and analgesia/steroids (paracetamol 1000 mg iv every 8 h; methylprednisolone 40 mg iv once daily), were used when required.

**Figure 3:**
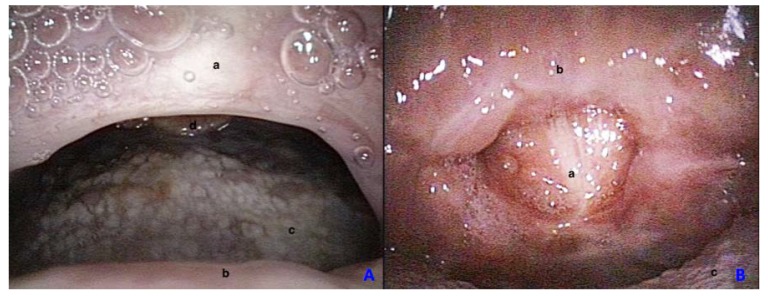
Post-operative control at 6 months from a nasopharyngeal view (**A**) and from an oral view (**B**). a—posterior pharyngeal wall, b—soft palate, c—tongue, d—epiglottis.

Postoperative follow up consists of an evaluation after three weeks, six months, and 2 year (mean follow-up time 24.2 months) (Figure 3).

### 2.4. Ethical Approval

All procedures performed were in accordance with the ethical standards of the institutional and/or national research committee and with the 1964 Helsinki declaration and its later amendments or comparable ethical standards. The study was approved by our institution ethics committee with n.AW24366751, 2014.

## 3. Results

Eight patients (mean age 27.25 years old (yo), range 8–67 yo; Male/Female ratio 4/4; mean body mass index (BMI) 26.1) who previously underwent pharyngeal surgery for OSA (mean AHI before OSA surgery was 22.1) and developed post-surgical nasopharyngeal stenosis were treated with our surgical palatoplasty variation to the one proposed by Toh et al. [8]. The surgical procedure that caused the pharyngeal obstruction differs among patients: adenotonsillectomy (four cases), tonsillectomy (one case), uvulopalatopharyngoplasty (two cases), and combined septoplasty-UPPP with coblator technique (one case). Only one patient was previously treated with surgical correction of the stenosis (2 interventions). All patients complained of pre-operative nasal respiratory obstruction. In three cases, snoring was referred and one patient complained of hypoacusis and fullness. Patients who underwent UPPP and combined surgeries (*n* = 3) presented with persistent OSA while the remaining patients showed AHI < 5.

Pre-operative VAS nasal breathing, dysphagia, dysphonia scores averages were respectively 6.75, 2.75, and 1.25.

During surgery, no patients had intra-operative complications.

At 3 weeks follow-up, only two patients complained of a nasal obstruction, one of them presented also with snoring. Mild dysphagia to solids and sore throat was reported by two patients. VAS nasal breathing, dysphagia, dysphonia scores averages were 2.62, 4.62, and 0.12, respectively.

After 6 months, only one patient reported persistent mild nasal respiratory obstruction. No postoperative hemorrhagic events, dysphagia, fibrotic re-stenosis >50% of the nasopharyngeal isthmus or palatal fistula were observed (Figure 3.). VAS nasal breathing, dysphagia, dysphonia scores averages were 0.

No complaints were referred by any patients at 2 years follow-up (All VAS scores were 0). No improvement in AHI was seen in patients with residual OSA. These patients continued Continous Positive Airway Pressure (CPAP) treatment successfully.

Results are summarized in Table 1.

## 4. Discussion

Taking into account the retrospective nature of our case series and its sample size, the following considerations can be made.

This study demonstrates that using this modified technique, the resolution of nasopharyngeal stenosis, as shown by complete reduction of patients’ symptoms, is seen at 2 years follow up. Persistence of OSA was observed in patients previously treated with UPPP and combined surgery with no improvement of polygraphy indices after our palatal procedure.

Several treatments for the correction of nasopharyngeal stenosis have been proposed in literature. However, not all treatments are surgical and their application depends on a variety of factors, such as the degree of the stenosis, patient’s age, and clinical condition. Among non-surgical treatments, local injection of triamcinolone acetonide has shown some success in treatment of mild pharyngeal stenosis, due to collagen dissolution and reducing the formation of keloids [10]. Nevertheless, surgical options are more effective and some of them include also minivasive procedures, such as reduction of pharyngeal scars by means plasma radio-frequency (Coblation) followed by mitomycin C injections [11].

In some cases, surgery can be performed under local anesthesia. Some authors opted to perform carbon dioxide scar lysis combined with the application of a nasopharyngeal obturator (worn from two to six months), local injection of corticosteroids, or followed by balloon dilatation [12] to reduce stenosis recurrences.

However, the best results seem to be obtained by the use of muscular-mucosal flaps especially in cases of severe stenosis: different flaps have been proposed with different localization in the pharyngeal region. One of the first techniques presented in the literature is the one described by Fritz [13], in which a nasal mucosal flap is used. Other flaps have been suggested such as the laterally based pharyngeal flap by Cotton [14] or the transposition of a facial artery musculomucosal (FAMM) flap by Wanjala Nangole [15]. However, one of the most used techniques is the one presented by Toh et al. [8], which implies the transposition of a palatal and a pharyngeal flap.

Pharyngeal flap surgeries should generally be preferred in cases of severe stenosis and may improve patients’ tolerance in comparison with nasopharyngeal obturators.

In all of these techniques, the most common complication is a re-stenosis after a first attempt of correction. In some cases, a velopharyngeal insufficiency with nasal reflux or palatal fistula may also occur.

From our experience, it seems that the proposed surgical technique, based on a nasopharyngeal mucosal flap splitting with a lateral suture of the two lateral flaps, could be considered a valid option for the treatment of acquired nasopharyngeal stenosis. This variation appears to be easy-to-perform, replicable, quick, and does not require any specific setting.

The main difference to Toh’s technique consists of the splitting of the palate flap into a right and a left flap. However, transposing the posterior half of the flaps anteriorly remains the common principle between the two surgical procedures.

In our opinion, our technique is less traumatic on muscular layers, and might reduce the risk of velopharyngeal insufficiency. However, Toh’s procedure can still be considered a preferable choice in cases of a complete stenosis, thus a customized approach for the treatment of this rare and variable condition is highly recommended.

In our series, all patients reported resolution of clinical symptoms with only one patient complaining of mild nasal obstruction at 6 months follow up. However, re-stenosis of the velopharyngeal opening has not been observed in our series. Moreover, post-operative palatal fistula, voice alteration, and moderate/severe nasal regurgitation have not been registered.

Our variation was also performed in four pediatric cases affected by moderate stenosis occurring after adenotonsillectomy, with a complete resolution of symptoms, showing a potential application in this specific pattern of patients.

However, further studies comparing different techniques and reporting on larger series and longer follow up time are needed to prove the efficacy of the proposed technique.

Unfortunately, the comparison of the efficiency of different techniques appears to be difficult to perform for several reasons. In fact, nasopharyngeal stenosis is a quite rare pathological entity and it presents a significant clinical variability often resulting in small and heterogeneous samples that hardly can support powerful comparative studies.

Finally, in our opinion, avoidance of respective surgeries such as UPPP and opting for more conservative, possibly cold steel, techniques such as barbed repositioning pharyngoplasty might help in the reduction of stenosis incidence [16].

## 5. Conclusions

Nasopharyngeal stenosis is a rare pathological post-operative entity, and adenotonsillectomy together with UPPP are the leading causes. The otolaryngologist has to be aware of this possible complication in order to decrease its incidence.

Taking into account the retrospective nature of our study and the size of our series, we believe that our technique appears an acceptable and promising method for the management of nasopharyngeal stenosis. However, further studies comparing different techniques and reporting on larger series and longer follow up time are needed to prove the efficacy of the proposed technique.

## Figures and Tables

**Table 1:** Table summarizing clinical data about the series.

Patient	Gender	Age(years)	Grade of Stenosis	Previous Surgery	Pre-Operative AHI	Pre-Operative VAS scores	Time Prior to Surgery (Months)	Hospitalization (Days)	3 Weeks VAS	6 Months VAS	2 Years VAS	2 Years Clinical Examination (Grade of Stenosis)	2 Years AHI
1	F	8	75%	adenotonsillectomy	2.6	nasal breathing 7 dysphagia 2 dysphonia 1	14	5	nasal breathing 2 dysphagia 5 dysphonia 0	nasal breathing 0 dysphagia 0 dysphonia 0	nasal breathing 0 dysphagia 0 dysphonia 0	25%	2.1
2	M	32	75%	Tonsillectomy	3.5	nasal breathing 7 dysphagia 2 dysphonia 0	16	4	nasal breathing 0 dysphagia 4 dysphonia 0	nasal breathing 0 dysphagia 0 dysphonia 0	nasal breathing 0 dysphagia 0 dysphonia 0	25%	3.2
3	M	54	50%	Uvulopalatopharyngoplasty	18.3	nasal breathing 6 dysphagia 3 dysphonia 0	14	6	nasal breathing 4 dysphagia 5 dysphonia 0	nasal breathing 0 dysphagia 0 dysphonia 0	nasal breathing 0 dysphagia 0 dysphonia 0	0%	17.6
4	F	23	75%	Uvulopalatopharyngoplasty	25.9	nasal breathing 7 dysphagia 2 dysphonia 0	13	6	nasal breathing 3 dysphagia 4 dysphonia 0	nasal breathing 0 dysphagia 0 dysphonia 0	nasal breathing 0 dysphagia 0 dysphonia 0	25%	26.3
5	F	67	50%	Uvulopalatopharyngoplasty + coblation And 2 interventions for stenosis correction	22.1	nasal breathing 6 dysphagia 4 dysphonia 2	32	4	nasal breathing 3 dysphagia 4 dysphonia 0	nasal breathing 0 dysphagia 0 dysphonia 0	nasal breathing 0 dysphagia 0 dysphonia 0	0%	24.1
6	M	13	75%	adenotonsillectomy	1.6	nasal breathing 8 dysphagia 4 dysphonia 3	24	4	nasal breathing 4 dysphagia 6 dysphonia 0	nasal breathing 0 dysphagia 0 dysphonia 0	nasal breathing 0 dysphagia 0 dysphonia 0	25%	1.9
7	M	9	75%	adenotonsillectomy	4.5	nasal breathing 6 dysphagia 3 dysphonia 1	14	5	nasal breathing 2 dysphagia 5 dysphonia 0	nasal breathing 0 dysphagia 0 dysphonia 0	nasal breathing 0 dysphagia 0 dysphonia 0	25%	3.4
8	F	12	75%	adenotonsillectomy	3.6	nasal breathing 7 dysphagia 2 dysphonia 2	27	6	nasal breathing 3 dysphagia 4 dysphonia 1	nasal breathing 0 dysphagia 0 dysphonia 0	nasal breathing 0 dysphagia 0 dysphonia 0	25%	2.7

Notes: Apnea Hypopnea Index (AHI), Visual Analog Scale (VAS).
